# Protective Properties of Spheroidal Taxifolin Form in Streptozotocin-Induced Diabetic Rats

**DOI:** 10.3390/ijms241511962

**Published:** 2023-07-26

**Authors:** Amir Taldaev, Anastasiya D. Savina, Vera V. Olicheva, Sergey V. Ivanov, Roman P. Terekhov, Igor R. Ilyasov, Anastasiya K. Zhevlakova, Irina A. Selivanova

**Affiliations:** 1 Laboratory of Nanobiotechnology, Institute of Biomedical Chemistry, Pogodinskaya Str. 10/8, 119121 Moscow, Russia; 2 Nelubin Institute of Pharmacy, Sechenov First Moscow State Medical University, Trubetskaya Str. 8/2, 119991 Moscow, Russia; 3 Laboratory of Psychopharmacology, V.V. Zakusov Research Institute of Pharmacology, 125315 Moscow, Russia

**Keywords:** taxifolin, diabetes mellitus, antioxidant properties, plasma antioxidant capacity, blood glucose

## Abstract

One of the key factors in the pathogenesis of diabetes and its complications is oxidative stress. To inhibit this process, antioxidants may be helpful. Herein, we focused on the protective properties of taxifolin spheroidal form (TS) in the streptozotocin rat model of diabetes mellitus. After 4 weeks of treatment with TS, the fasting blood glucose level of the diabetic animals decreased by 12% compared with the level right after the injection of streptozotocin. While the feed intake in the untreated diabetic rats increased by 5.3% compared with the healthy group, the TS-treated group showed a pronounced 15.3% decrease. Therapeutic administration of TS has a protective effect on the pancreas and the liver against the cytotoxic action of streptozotocin. The plasma antioxidant capacity of all diabetic groups appeared to be approximately 15% lower than in healthy rats with no significant difference between the TS-treated and untreated diabetic animals. Apparently, this can be attributed to taxifolin and plasma proteins binding. These data demonstrate the potential of TS in antidiabetic therapy.

## 1. Introduction

Diabetes mellitus (DM) is a disease that affects a large number of people all over the world. The total number of patients with this pathology increases year after year. According to the International Diabetes Federation, 537 million adults are living with diabetes. This number is predicted to rise to 643 million by 2030 and 783 million by 2045 [1]. On average, death occurs at the age of 55 [2]. During the first 5–6 years of development of prediabetes and diabetes mellitus type 2 (DM2), a person may not know about the diagnosis itself and the macro- and microvascular complications it leads to [3].

One of the main pathogenetic factors of DM is oxidative stress. The accumulation of peroxide, alkyl, alkoxyl radicals, superoxide anion, singlet oxygen, and cytotoxic peroxynitrites, and their reactions with membrane phospholipids, cause their structural changes and cell death [4,5].

The development of DM is characterized both by the increase of free radical formation and the decrease of antioxidant capacity of endogenous enzymes, such as superoxide dismutase, catalase, glutathione peroxidase, etc. [6]. Excessive generation of free radicals leads to activation of protein kinases, increased glycosylation of proteins, disruption of intercellular connections, damage to signal transduction, and accumulation of NADP·H, which causes further proinflammatory processes and cell death [7]. It is important to emphasize that in pancreatic β-cells the deficiency in antioxidant systems and the accumulation of free radicals are more pronounced than in other organs [8]. Therefore, the study of substances with antioxidant activity is one of the promising areas of the search for new antidiabetic drugs.

Taxifolin (also known as dihydroquercetin (2,3-dihydro-3,5,7-trihydroxy-2-(3,4-dihydroxyphenyl)-4*H*-1-benzopyranone-4)) is a naturally-occurring flavonoid, which is industrially obtained from the wood of Dahurian larch (*Larix dahurica* Turcz.) as an active pharmaceutical ingredient in Russia. It also presents in the European market of food supplements. This compound demonstrates anti-inflammatory [9], antitumor [10], antiviral [11,12], capillary-protective [13,14], hepatoprotective [15,16], neuroprotective [17,18], and regenerative [19] activities.

Taxifolin can exist in several phase modifications [20,21,22]. The change of solid state may result in improvement in oral bioavailability [23], dissolution behavior [24], and activity [25] of the pharmaceutical ingredient. The crystal modification of taxifolin served as the basis for the synthesis of taxifolin spheroidal form (TS) via spray drying [26]. The advantages of TS are enhanced water solubility at common conditions, a higher safety profile, and a prolonged release mode from tablets [27]. Therefore, TS may be considered a preferred form for long-term therapy.

Thus, we aimed to study the effect of TS on the streptozotocin (STZ) model of diabetes mellitus type 1 (DM1) in Wistar rats.

## 2. Results

### 2.1. General Outcomes

The survival rate in the untreated group was 85.7%, while in the healthy and treated groups all rats survived.

The results of body weight monitoring are presented in Table 1. The initial body weight did not differ in all groups with an average of 283.1 ± 7.5 g. The body weight of healthy rats increased gradually and became generally stable at day 14. The untreated group showed no significant weight change through all 28 days. At the same time, treated rats demonstrated significant weight loss by day 7 and then were mainly unchanged. By day 28, this parameter significantly differed between groups with a maximum of 355 g for the healthy group and a minimum of 211 g for treated rats.

Food intake monitoring is shown in Table 2. Obviously, there was not a significant difference in food intake for each group week by week. The difference between healthy and untreated groups was not observed; however, untreated and treated groups differed significantly. Additionally, on day 7, there was some difference between healthy and treated groups, which became insignificant by day 21.

### 2.2. Blood Glucose Monitoring

**Figure 1:**
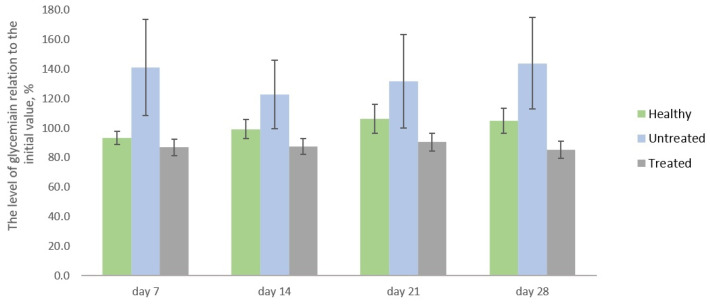
Relative changes in glycemia level in relation to day 1.

After STZ injection the fasting blood glucose level increased up to 18.8 ± 1.8 mmol/L and 25.5 ± 0.9 mmol/L for untreated and treated groups, respectively, from 6.0 ± 0.3 mmol/L in healthy rats. Then, in the untreated group, glycemia achieved 23.4 ± 2.1 mmol/L (+41.1%) on day 7 with no further changes. Conversely, in the treated group this parameter decreased down to 21.4 ± 1.0 mmol/L (−14.7%) by day 28. In healthy rats, the glucose concentration was generally constant. The dynamics of changes are reflected in Figure 1.

**Figure 2:**
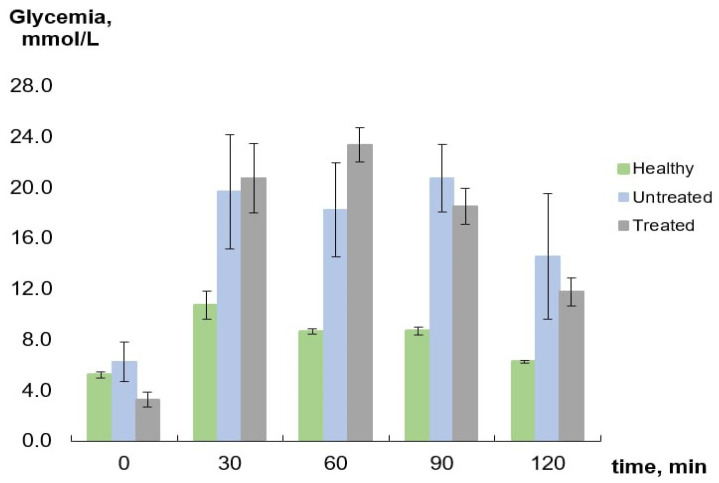
OGTT results.

The oral glucose tolerance test (OGTT) in healthy animals showed that, in response to a glucose load, the glycemic level reached a maximum of 10.7 ± 1.1 mmol/L after 30 min and returned to the basal level after 120 min. At the same time, in the untreated group, the level of glycemia reached 19.7 ± 4.5 mmol/L with no further changes by 120 min. In the treated group, the blood glucose level grew up to 20.7 ± 2.8 mmol/L by 30 min, remained unchanged for the next 30 min, and then gradually decreased down to 11.8 ± 1.1 mmol/L by 120 min (Figure 2).

### 2.3. Histology Analysis

**Figure 3:**
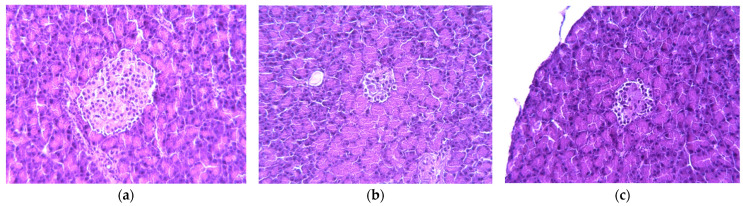
Micrographs of histological preparations of the pancreas in different experimental groups at 600× magnification: (**a**) healthy; (**b**) untreated; (**c**) treated with TS. Hematoxylin-eosin.

During histology analysis, all pancreas samples demonstrated a lobular structure with exocrine and endocrine parts (Figure 3). The pancreatic acini are located close to each other and pass into the excretory inset and striated ducts. Islets of Langerhans have an oval elongated shape; however, in diabetic animals, their number is reduced. For untreated rats in the exocrine part of the pancreas, a greater number of oxyphilic granules (zymogen, an inactive enzyme) was detected in the apical pole of cells than in the healthy group. These endocrine cells in the central part of the Langerhans islets have signs of moderate hypertrophy. The pathology intensified by blood vessels damages with the replacement of connective tissue and swelling of the walls.

Therapeutic administration of TS to diabetic rats showed a protective effect on the pancreas that results in a decrease in atrophy area, normalization of the islets’ morphology, enlargement of the islets of Langerhans, and clean blood vessels without signs of damage.

**Figure 4:**
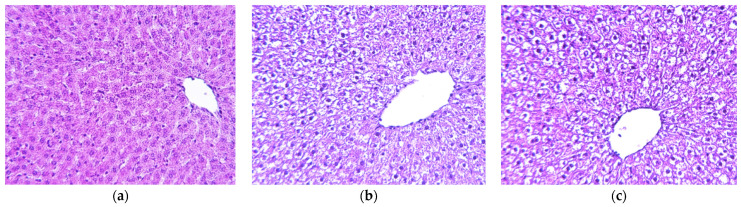
Micrographs of histological preparations of the liver in different experimental groups at 600× magnification: (**a**) healthy; (**b**) untreated; (**c**) treated with TS. Hematoxylin-eosin.

Turning to the liver islets, the healthy rats showed histoarchitectonics of the lobular structure with the vein in the center of lobules from which the hepatic beams with sinusoids radially alternate. The hepatic beam is formed by two or three rows of polygonal or cubic hepatocytes with a centrally located large round nucleus. The interlobular septa are thin, formed by loose unformed fibrous connective tissue. The interlobular blood and lymphatic vessels and the bile capillary form a triad and tetrad at the vertices of the classical hepatic lobule (Figure 4a).

In diabetic rats, the beam histological structure of the liver is slightly impaired due to hepatic steatosis of a diffuse nature (Figure 4b,c). The fatty degeneration of hepatocytes with the infiltration and transformation as main development mechanisms is observed. About 2/3 of hepatocytes located mainly in the perivenular zone of the hepatic lobule in untreated and 1/3 in treated groups are large, some are vacuolated, and their cytoplasm contains pulverized lipid inclusions. The detected pathomorphological changes lead to functional disorders of the structural units of the portal lobule and hepatic acinus. In addition, there is moderate edema in the liver parenchyma in treated rats.

### 2.4. Antioxidant Activity of Plasma

**Figure 5:**
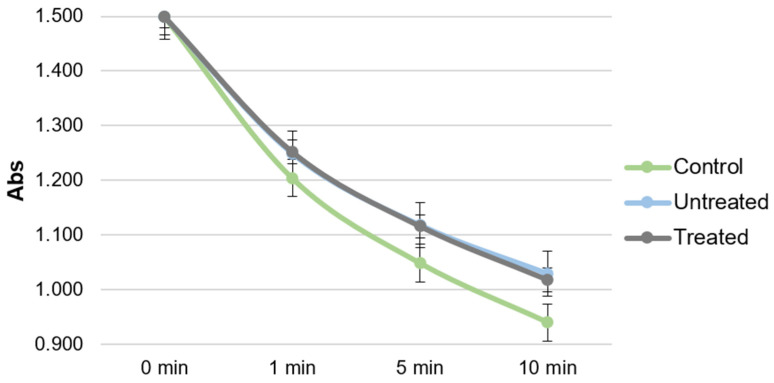
Plasma absorption comparison of different groups.

The absorbance loss due to the ABTS^•+^ self-bleaching appeared to be 0.04 ± 0.01 by 10 min. Incubation with rat plasma samples led to a substantial decrease in the ABTS^•+^ absorption with mean ∆Abs 0.56 ± 0.03, 0.47 ± 0.04, and 0.48 ± 0.02 for healthy, untreated, and treated rats, respectively (Figure 5). When TS was incubated for 3 h with healthy rat plasma to model its binding to plasma proteins, the antioxidant capacity of plasma changed from ∆Abs 0.79 ± 0.04 without to 1.19 ± 0.09 with the addition of TS. The observed ∆Abs values of the rat plasma with and without TS and TS itself are presented in Table 3. The results of TS testing at the initial ABTS^•+^ absorbance of 0.85 demonstrated full inhibition of ABTS^•+^ radicals.

## 3. Discussion

This study was performed to evaluate the effects of TS on DM1 in Wistar rats using STZ in the dose of 45 mg/kg for pathology modeling. In this case, the glycemia formation is associated with the decrease of insulin blood concentration due to the death of cells in the Langerhans islets. However, as was shown before, 45 mg/kg STZ leads to a decrease in the level of insulin in the blood by 48% and a viability of about 30% beta cells in the pancreas which correlates with the clinical pattern of DM2 [28,29]. Thus, the STZ-induced DM is not a strict model and characterized by signs of both insulin-dependent and insulin-independent types.

Kondo et al. investigated taxifolin as an antidiabetic agent and showed a decrease in the level of glycated hemoglobin. Research on a small sample of mice demonstrated improvement in glycemic control and an index of insulin resistance [30]. It is important to note that this research was performed in the mouse model of DM2, which is a common practice but does not accurately fit the context of DM1 in rats. Furthermore, it was tested with the crystal form of taxifolin. Previously, we showed that the different phase modification of taxifolin may differ significantly in pharmacological efficacy [31]. Therefore, it was of interest to study the TS form against DM1.

Our results are in line with these data. Even though we showed that treatment with TS 50 mg/kg/day administrated orally results in a decrease of 12.4% in fasting blood glucose level compared to the initial level after 4 weeks, which is not that considerable, the OGTT demonstrated the improvement of glucose tolerance by the day 28, the result which was not observed for untreated rats.

During the histological analysis, the pancreas sections of healthy animals showed normal pancreatic acini and Langerhans islets. The islets have a regular oval shape with clear contours, without signs of destruction and inflammatory elements. In contrast, the STZ injection led to sufficient damage of pancreas cells characterized by a change in shape and size, hypertrophied nuclei, as well as lymphoid and macrophage infiltration. TS treatment has a protective effect on the pancreas under STZ cytotoxic action that results in the reduction of inflammatory elements and restoration of the morphological structure of the islet. To our knowledge, it is the first time the histological analysis of taxifolin action on the pancreas in DM1 is reported.

The hepatoprotective action of TS 50 mg/kg/day manifested as a sufficient decrease in the number of affected hepatocytes in the treated group, two times less than in the untreated group. The balloon dystrophy and fibrosis severity were less pronounced than in the untreated diabetic group. The hepatic lobular and bar structure was preserved. A similar effect was observed by Teselkin et al. in tetrachloromethane-induced hepatitis in rats where this effect was attributed to taxifolin antioxidant properties [16].

**Figure 6:**
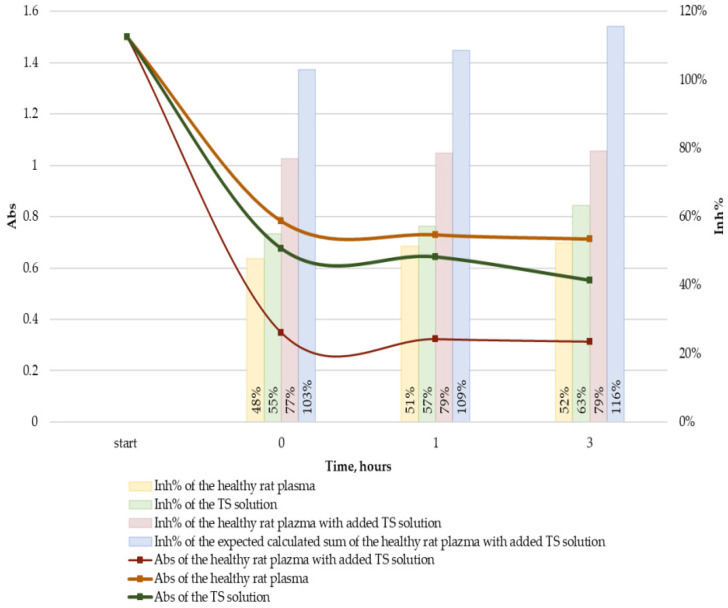
Kinetic curves of the inhibition of ABTS^•+^ radicals by samples of healthy rat plasma, TS solution, and healthy rat plasma with added TS solution: without preliminary incubation, and after 1, or 3 h of incubation at 37 °C. Clustered columns chart shows the percent of ABTS^•+^ inhibition (Inh%) by the abovementioned samples at the same time points.

The comparison of the antioxidant capacity for plasma samples of healthy rats with all diabetic rats demonstrated a significant, approximately 15% decrease in the case of diabetic ones. In general, the decreased antioxidant capacity of blood samples both in the treated and untreated groups in comparison with healthy rats was associated with a considerable increase in the fasting blood glucose levels and a two-fold sharper rise in the 30-min blood glucose levels in OGTT. This result was expected between the groups of healthy rats and untreated diabetic rats. At the same time, we anticipated finding some difference between untreated diabetic rats and TS-treated rats but failed to see any difference. The possible reason for this is that the TS addition effect is masked by its binding to proteins. To check this hypothesis, we tested the antioxidant capacity of healthy rats’ plasma with and without the addition of TS and compared it with TS itself. The concentration of TS in plasma was such as to model its 100% bioavailability in our conditions, 2.1 mmol/L. The observed plasma with TS ∆Abs values appeared to be approximately 30% less than the expected calculated value (see Table 2, Figure 6). This effect could be the result of the TS binding to proteins; however, it could also be attributed to the difference in the ABTS^•+^/antioxidant ratio: Our doubts were the result of an assumption that when TS is measured itself it can inhibit ABTS^•+^ deeper as all the ABTS^•+^ reacts to it solely thus the range of the possible reaction pathways can be wider [32]. At the same time when plasma copresents in the incubation mixture it inhibits almost half of ABTS^•+^ itself, so only another half is supplied to TS. To dismiss this hypothesis, we tested TS not only at the initial 1.5 ABTS^•+^ absorbance as in all other experiments, but also at the reduced 0.85 ABTS^•+^ initial absorbance, modeling the loss of 0.65 absorption because of plasma antioxidants. The observed full inhibition of ABTS^•+^ radicals by TS in these conditions dissipated all the doubts. Thus, it was the effect of the antioxidant capacity masking due to the interaction of flavonoids with proteins that have already been described previously and apparently played a substantial role in our study too [33].

One of the most interesting and unexpected results of our research was the considerable weight loss in the treated group. Only in the untreated group, the body weight stayed generally the same. The healthy rats gained more than 50 g weight which, to our careful consideration, is quite common. The treatment with TS 50 mg/kg/day led to a dramatic weight loss of about 22% from 283 g to 221 g during the first week. The weight loss was accompanied by a change in eating behavior. To the best of our knowledge, this phenomenon was not reported previously. The reason for the observed phenomena is unclear and further investigations are needed. Nevertheless, it is important to note, that insulin is an anabolic hormone, and DM1 is associated with a decrease in body weight [34]. Perhaps the rats in the treated group were more sensitive to the toxic effect of STZ, which results in more severity of weight loss. However, the disease model cannot explain hypophagia formation, while polyphagia is specific for DM [35,36]. In any case, it is unlikely that this may be related to the taste characteristics of TS, since the introduction was directly into the pharynx, bypassing the taste buds. All in all, it somehow affects the appetite of rats and therefore is promising from the point of view of antiobesity therapy.

We are well aware of the pragmatic nature of our research and the possible limits of our results, which were obtained not in a double-blind study. For these reasons, we assume these results to be carefully confirmed under more controlled conditions and on larger cohorts of animals. In addition, it is worth noting that our study did not include monitoring several key markers of diabetes and its complications, including glycated hemoglobin and insulin in the blood. In light of these considerations, we view our results as a necessary starting point for a new path that must be taken to verify our results beyond any reasonable doubt.

## 4. Materials and Methods

### 4.1. Animals

The adult male Wistar rats with an initial body weight of 270–290 g were obtained from Stolbovaya Nursery (Stolbovaya, Russia). The animals were given free access to food and water, except for 16 h before injection of STZ and conducting the OGTT and fasting blood glucose when food was excluded. The animals were kept in a temperature and humidity-controlled room in accordance with Appendix A of the European Convention for the Protection of Vertebrate Animals used for experimental and other scientific purposes (ETS N 123) Guidelines for accommodation and care of animals. The experimental protocols were approved by a local Ethical Committee of V.V. Zakusov Research Institute of Pharmacology (Moscow, Russia).

### 4.2. Materials

Taxifolin was purchased from JSC Ametis (Blagoveshchensk, Russia) and then processed according to spray drying technology, reported previously [27], to obtain TS. STZ, 2,2′-azinobis(3-ethylbenzothiazoline-6-sulfonic acid) diammonium salt (ABTS, ≥98% HPLC grade), potassium persulfate (di-potassium peroxydisulfate, PP), phosphate buffer solution (PBS, pH 7.4), 10% buffered formalin (pH 7.4), and sodium chloride were obtained from Sigma-Aldrich (Burlington, MA, USA). Citrate buffer (pH 4.5) was obtained from JSC COMPONENT-REAKTIV (Moscow, Russia). Saline solution (SS) from Avexima Siberia (Anzhero-Sudzhensk, Russia), the HPLC grade ethanol from Merck Ltd. (Darmstadt, Germany), and the deionized water from LLC Smoly (Moscow, Russia) were used as solvents.

### 4.3. Study Design

DM1 was induced via a single intraperitoneal injection by using the fresh 4.5% solution of STZ in cold citrate buffer to achieve the dose of 45 mg/kg, taking into account the previous findings [28]. The experiment included animals whose blood glucose level after 72 h of the STZ injection was at least 15 mmol/L.

**Figure 7:**
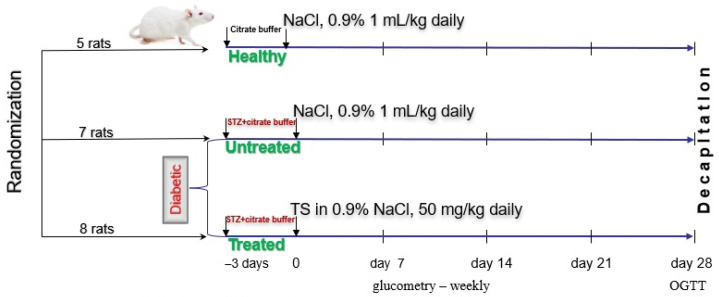
The study design (TS is spheroidal taxifolin form).

Rats (*n* = 20) were randomized into three groups (Figure 7). The healthy group (*n* = 5) on the 1st day of the experiment was treated with citrate buffer via a single intraperitoneal injection, and during the next 28 days SS in a volume of 1 mL/kg/day was administrated orally. Diabetic rats of the untreated group (*n* = 7) on the 1st day of the experiment were injected with STZ 45 mg/kg, and during the next 28 days SS in a volume of 1 mL/kg/day was administrated orally. Diabetic rats of the treated with TS group (*n* = 8) on the 1st day of the experiment were injected with STZ 45 mg/kg, and during the next 28 days TS suspension in SS was administrated orally in a dose of 50 mg/kg.

For each group, the consumption of water and feed was fixed daily, while the body weight was measured weekly. The fasting glucose level was determined using the One Touch Select Plus Flex blood glucose monitoring system (LifeScan, Zug, Switzerland) on day 0, day 7, day 14, day 21, and day 28 of the experiment (see Figure 1). To obtain the average level of glycemia relative to the initial value (*N*, %) the Formula (1) was used for each group:

(1) $$N=\frac{\sum_{i=1}^{a}\left(\frac{n_{x}}{n_{0}}\right)}{a}\times 100\%,$$

where *n*_0_ is the initial concentration of glucose in the blood of an individual animal (mmol/L), *n_x_* is the concentration of glucose in the blood of an individual animal on the day of measurement (mmol/L), *a* is the number of animals in a group.

On day 29, the animals were decapitated under ether anesthesia. Blood was collected in tubes with EDTA and centrifuged for 3 min at 3000 rpm; plasma was stored at −20 °C. To measure the antioxidant capacity of plasma samples, they were thawed before the analysis and then added to the incubation mixture in the ABTS/PP assay in the amount of 10 µL without further dilution.

### 4.4. OGTT

On day 28, an OGTT was performed: Glucose (3.0 g/kg) was administered orally following 16 h fasting with free access to water. Blood samples were collected from the tail vein just before (0) and 30, 60, 90, and 120 min after glucose administration. All blood glucose measurements were performed using the One Touch Select Plus Flex blood glucose monitoring system (LifeScan, Zug, Switzerland).

### 4.5. Pancreas and Liver Morphological Analysis

Organs were fixed in 10% buffered formalin, underwent processing for 24 h, dehydrated, and then embedded in paraffin. The paraffin sections were cut into 2 µm thick slides by a rotary microtome (Microtome Leica RM2265, Germany). They were placed in a warm water bath to spread the paraffin. After that, the slices were transferred to a slide and placed on the Leica HI 1220 heating table (Nussloch, Germany). Then, the slides were stained with hematoxylin and eosin according to the manufacturer’s standard protocol (BioVitrum, St. Petersburg, Russia) using Leica Autostainer XL (Nussloch, Germany). Then, they were put in synthetic mounting medium Leica SUB-X (Nussloch, Germany) for coverslipping operation on Leica CV5030 robotic cover slipper (Nussloch, Germany). Micropreparations were analyzed using a Leica DM2000 microscope (Leica Microsystems, Wetzlar, Germany) with a ToupCam UCMOS03100KPA digital camera (Hangzhou, China). Magnification was 600×.

### 4.6. Measurement of Antioxidant Activity of Plasma

The antioxidant capacity of plasma samples with or without TS was assayed by employing the slightly modified ABTS/PP method [37,38,39]. In short, this approach is based on the inhibition of ABTS^•+^ radicals by antioxidants. The ABTS^•+^ radicals are rather stable, and their concentration is easy to monitor spectrophotometrically at specific absorption maxima, usually 412 or 730 nm. To obtain the stock ABTS^•+^, the solutions of ABTS and PP were mixed at final concentrations of 7 mM and 2.5 mM the day before analysis and put in a dark place to react. To assess the antioxidant capacity of plasma samples with or without TS, they were added to the solution of ABTS^•+^ in PBS (pH 7.4), shaken intensively for 15 s, and then put in the dark heat chamber at 37 °C. The ABTS^•+^ absorption at 730 nm was monitored right before and 10 min after the addition of the tested samples. The decrease in the ABTS^•+^ absorption during incubation without antioxidant samples was also measured at the same time points to take ABTS^•+^ self-bleaching into account. The initial ABTS^•+^ absorbance in the incubation mixture before the addition of an antioxidant was adjusted to 1.5 ± 0.1 for all experiments except for TS testing, where measurements were held both at the initial 1.5 ± 0.1 and 0.85 ± 0.1 ABTS^•+^ absorbance. All the UV-Vis spectra were obtained on Varian Cary 100 spectrophotometer, pathlength 1 cm.

The absorbance loss (∆Abs) at 730 nm was calculated to compare the antioxidant capacity of the samples considering the initial absorbance A_initial_, the absorbance after 10 min of incubation A_10min_, and the decrease due to the self-bleaching ∆A_self-bleaching_, as follows (Formula (2)):

(2) $$∆Abs=A_{initial}-A_{10\;min}-{∆A}_{self-bleaching},$$

The percent of ABTS^•+^ inhibition (Inh%) by the sample in the incubated mixture was calculated according to the next Equation (3):

(3) $$Inh\%=\frac{∆Abs}{A_{initial}}\times 100\%,$$

All solutions of the reagents were prepared on the day of use. A stock solution of TS was dissolved in ethanol to obtain the working solutions, which were then added to the incubation mixture or added to the plasma samples. ABTS and PP were dissolved in deionized water. None of the solvents interfered with the assays.

### 4.7. Modeling TS Binding to Plasma Proteins

Two samples of plasma from a healthy rat were taken 0.2 mL each. Ten microliters of the stock 44 mM TS solution in ethanol were added to the first sample to the final concentration of 2.1 mM. The second sample was a reference control, so the same amount of ethanol was added. The 10 µL aliquots of both samples were taken for the antioxidant capacity testing forthwith. The rest parts of both samples were put in an Eppendorf thermostat 5320 (Hamburg, Germany) at 37 °C to repeat antioxidant capacity testing 1 h and 3 h later. TS itself was also measured in the same concentration and conditions as a reference.

### 4.8. Statistics

The results of the measurements are shown as the mean (±standard deviation) of at least three determinations. The paired Student’s *t*-test was used for the analysis of experimental results and *p* < 0.05.

## 5. Conclusions

This project is the first comprehensive investigation of TS in DM. The observed results supported our suggestions that TS helps moderately to improve the glucose level during DM1 in rats. Despite a modest fasting blood glucose level decrease, the OGTT results were promising. Finally, we observed considerable protective effects on pancreatic and liver tissues.

The obtained findings encourage us to the follow-up efforts directed to the pharmaceutical development of a dosage form based on TS and the study of its therapeutic window.

## Figures and Tables

**Table 1:** Monitoring of body weight.

Group	Body Weight, g				
	Day 0	Day 7	Day 14	Day 21	Day 28
Healthy	291.6 ± 4.9	327.4 ± 8.3	341.6 ± 6.1	354.4 ± 6.8	355.0 ± 6.5
Untreated	276.7 ± 10.2	248.6 ± 19.5	248.1 ± 24.8	258.3 ± 27.5	252.8 ± 26.2
Treated	283.3 ± 6.9	220.9 ± 5.6	212.8 ± 7.6	213.1 ± 10.0	211.3 ± 9.4

**Table 2:** Monitoring of the rats’ eating behavior.

Group	Food Intake, g/Day/Rat				
	Day 0	Day 7	Day 14	Day 21	Day 28
Healthy	23.2 ± 0.9	26.9 ± 1.8	25.6 ± 1.4	23.4 ± 3.0	27.9 ± 3.1
Untreated	23.4 ± 1.5	25.9 ± 1.4	26.3 ± 1.4	27.5 ± 1.2	29.6 ± 2.3
Treated	23.3 ± 1.2	20.6 ± 2.1	21.9 ± 0.9	23.5 ± 1.0	21.9 ± 3.0

**Table 3:** Results of the modeling of TS binding to plasma proteins: ∆Abs values for the samples tested after preliminary incubation at 37 °C.

Time, h	∆Abs Values				The Observed Value Difference from the Expected Value, %
	Healthy Rat Plasma	TS Solution	Healthy Rat Plasma with Added TS Solution		
			Observed	Expected	
0	0.72 ± 0.08	0.83 ± 0.01	1.15 ± 0.05	1.54 ± 0.09	−25 ± 0 *
1	0.77 ± 0.03	0.86 ± 0.02	1.18 ± 0.02	1.63 ± 0.05	−28 ± 1 *
3	0.79 ± 0.04	0.95 ± 0.05	1.19 ± 0.09	1.74 ± 0.09	−32 ± 2 *

* Statistically significant with *p* ≤ 0.05.

## Data Availability

Data is contained within the article.
